# SGMR-LPR: A Semantic-Guided Network Robust to Movable Objects for LiDAR-Based Place Recognition

**DOI:** 10.3390/s26103050

**Published:** 2026-05-12

**Authors:** Weizhong Jiang, Zhipeng Xiao, Lilin Qian, Erke Shang, Dawei Zhao, Qi Zhu, Liang Xiao

**Affiliations:** Defense Innovation Institute, Beijing 100071, China; jiangweizhong16@alumni.nudt.edu.cn (W.J.); qianlilin15@nudt.edu.cn (L.Q.); shangerke10@alumni.nudt.edu.cn (E.S.); zhaodawei12@alumni.nudt.edu.cn (D.Z.); xiaoliang@nudt.edu.cn (L.X.)

**Keywords:** 3D point cloud processing, LiDAR-based place recognition, scene understanding, movable objects robustness, semantic guidance, BEV feature modulation, attention mechanism

## Abstract

**Highlights:**

**What are the main findings?**

- We propose SGMR-LPR, an end-to-end semantic-guided framework that explicitlymodels and suppressesmovable-object interference in LiDAR-based place recognition through a probabilistic movable object masking (PMOM) module, which converts semantic logits into uncertainty-aware continuousmasks to softly encodemovable regions.
- We introduce a movable-suppressed channel–spatial attention (MSCS) module that leverages these probabilistic masks to adaptively modulate high-level BEV (Bird’s Eye View) features—attenuating responses from movable-object areas while enhancing structurally stable scene elements—resulting in robust place representations under dynamic scene variations.

**What are the implications of the main findings?**

- By embedding explicit movable-awareness directly into feature modulation without requiring external semantic models or annotations at inference, SGMR-LPR provides a practical and deployable solution for reliable loop closure and re-localization in autonomous systems operating in traffic-intensive urban environments.
- The proposed probabilisticmasking and attention-based suppression paradigmadvances point cloud intelligence by demonstrating that soft, uncertainty-aware handling of movable objects is more effective than hard filtering or implicit regularization, offering a new direction for robust scene understanding under real-world dynamic conditions.

**Abstract:**

Robust LiDAR point cloud processing in dynamic outdoor environments, where movable objects such as vehicles and pedestrians introduce significant structural uncertainty, remains a key challenge for remote sensing and autonomous systems. This work addresses LiDAR-based place recognition (LPR), a critical component for loop closure and re-localization that is highly susceptible to such dynamics. While semantic information is beneficial, existing methods often require external segmentation models at inference or lack explicit mechanisms to suppress movable objects under uncertain predictions. To address these limitations, we propose SGMR-LPR, an end-to-end semantic-guided framework designed to explicitly counteract movable-object interference during feature encoding. Building on the “segmentation-while-describing” paradigm, SGMR-LPR incorporates an internal semantic segmentation branch and two novel modules: a probabilistic movable object masking (PMOM) module, which transforms semantic logits into continuous, uncertainty-aware masks of movable regions; and a movable-suppressed channel–spatial attention (MSCS) module, which uses these masks to adaptively modulate high-level BEV features—suppressing responses from movable-object regions while enhancing stable structural elements. By embedding explicit movable-awareness into feature modulation, SGMR-LPR achieves enhanced robustness without external semantic models at inference. Extensive experiments on multiple benchmarks demonstrate consistent performance gains, particularly in scenes with dense movable objects, advancing reliable point cloud-based scene understanding in dynamic environments.

## 1. Introduction

The intelligent processing of 3D LiDAR point clouds is pivotal for advancing scene understanding and modeling in applications such as autonomous navigation, urban digitization, and environmental monitoring. A fundamental challenge in this domain is the inherent dynamism of real-world environments, where movable objects (e.g., vehicles, pedestrians) introduce non-stationary elements that corrupt the structural consistency of point cloud data. Developing robust algorithms to distill reliable representations from such dynamic scenes is therefore a central pursuit in point cloud intelligence.

As a critical component for achieving long-term spatial consistency in dynamic environments, place recognition (PR) aims to determine whether a current sensor observation corresponds to a previously visited location by matching against a map or database. It plays a critical role in loop closure detection and relocalization within SLAM (Simultaneous Localization and Mapping) systems, helping to correct accumulated localization drift and maintain long-term localization reliability [1]. According to the sensing modality, PR methods are commonly categorized into Visual-based Place Recognition (VPR) and LiDAR-based place recognition (LPR). Compared with camera-based approaches that are sensitive to illumination, weather, and seasonal variations, LiDAR provides accurate geometric measurements with long sensing range and strong robustness to appearance changes, making LPR particularly suitable for large-scale outdoor scenarios [2].

**Figure 1:**
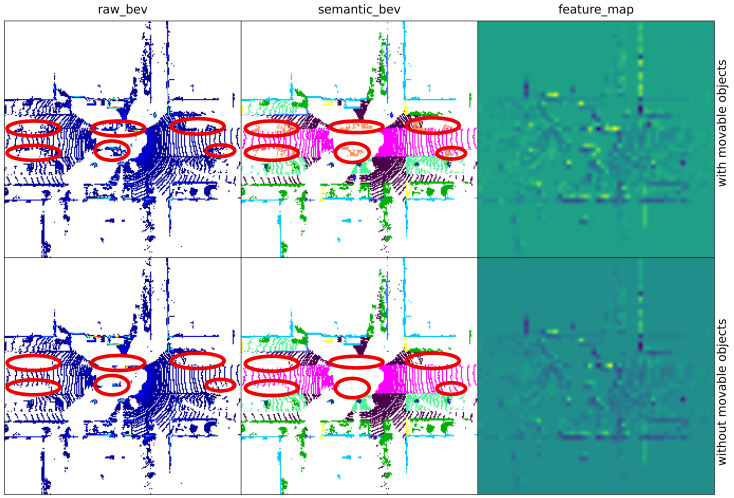
Visualization of the influence of movable objects on BEV observations and the corresponding feature representations at the same physical location. The first row presents BEV representations in the presence of movable objects, including the raw BEV, the semantic BEV, and the corresponding backbone feature map. The second row shows the BEV representations after movable objects are removed at the same location. The feature maps in the third column are extracted from the backbone network without any movable-object suppression. A clear discrepancy can be observed between the two feature maps at identical spatial positions, demonstrating that movable objects significantly perturb the encoded representations and consequently degrade the robustness of LPR. The red ellipses indicate the regions affected by movable objects in both the raw BEV and the semantic BEV, highlighting the local region changes before and after movable-object removal in the first and second rows, respectively.

Despite these advantages, the robustness of LPR is severely challenged by dynamic environments, where movable objects such as vehicles and pedestrians frequently appear, disappear, or change positions across revisits. Unlike static structures (e.g., buildings or road layouts), movable objects may be temporarily stationary during data acquisition but are inherently unstable over time. Consequently, LiDAR point clouds captured at the same place can exhibit significant structural discrepancies due to the presence or absence of movable objects. Since most LPR methods rely on the structural consistency of scenes to construct discriminative place representations, such variations often lead to false matches or missed detections, especially in traffic-intensive urban environments [3]. Figure 1 provides a visual illustration of how movable objects significantly alter BEV observations and the resulting feature responses at the same place.

To alleviate the adverse influence of scene dynamics, semantic information has been widely explored as an effective cue for improving LPR robustness [4,5]. By distinguishing different object categories, semantic-aware LPR methods can better focus on structurally stable elements while down-weighting semantically unstable regions. However, most existing semantic-based approaches either depend on handcrafted semantic representations [6] or require semantic predictions from independently trained segmentation models during inference [7,8,9]. These designs not only increase system complexity but also introduce additional uncertainty due to error propagation across loosely coupled modules, making end-to-end optimization and deployment more challenging in real-world dynamic environments [10].

An alternative direction is the “segmentation-while-describing” paradigm, exemplified by SG-LPR, which incorporates a semantic segmentation task as an auxiliary objective during training to guide the backbone network toward learning semantically meaningful representations. By removing the segmentation branch at inference time, such methods avoid the need for external semantic models during deployment [10]. While this paradigm successfully injects high-level semantic cues into the feature learning process, the influence of the segmentation task remains largely implicit. In particular, the learned place representations are not explicitly modulated according to different semantic regions, and features corresponding to movable objects may still contribute to the global descriptor. As a result, the inherent instability of movable objects is not directly addressed, which can limit robustness in highly dynamic driving scenarios.

Motivated by the above observations, we propose SGMR-LPR, an end-to-end semantic-guided movable-robust LPR framework that explicitly models and suppresses the influence of movable objects during feature encoding. Building upon the “segmentation-while-describing” paradigm, SGMR-LPR establishes a direct functional connection between the semantic segmentation auxiliary task and the LPR main task. Instead of treating semantic supervision merely as an implicit regularizer, semantic predictions are explicitly exploited at inference time to guide feature modulation in the LPR branch, without introducing external semantic models or additional semantic annotations.

Specifically, SGMR-LPR introduces a probabilistic movable object masking (PMOM) module, which converts semantic logits produced by the segmentation branch into probabilistic movable-object masks through soft semantic encoding. These masks provide continuous estimates of the likelihood that each spatial location corresponds to a movable object, naturally accounting for uncertainty in semantic predictions. Guided by the generated masks, a movable-suppressed channel–spatial attention (MSCS) module is designed to adaptively modulate high-level BEV features by suppressing responses from regions with high movable-object probability while enhancing structurally stable scene elements via joint spatial and channel attention. Through this explicit movable-aware feature modulation mechanism, SGMR-LPR effectively reduces the detrimental impact of movable objects and improves place recognition robustness in high dynamic outdoor environments.

The main contributions of this paper can be summarized as follows:

- We explicitly address the challenge of movable-object interference in LiDAR-based place recognition by introducing a semantic-guided movable-robust framework that goes beyond implicit semantic regularization.
- We propose a probabilistic movable object masking (PMOM) mechanism that softly encodes semantic predictions into uncertainty-aware movable-object masks, enabling robust suppression under imperfect semantic estimation.
- We design a movable-suppressed channel–spatial attention module that leverages probabilistic movable-object masks to modulate high-level BEV features, enhancing robustness to dynamic scene variations.
- Extensive experiments on multiple benchmark datasets demonstrate that SGMR-LPR consistently outperforms existing methods, particularly in environments with dense movable objects.

## 2. Related Work

This section systematically reviews existing LPR research, focusing on three core directions: non-semantic LPR methods, semantic-guided LPR methods, and movable object handling methods. It clarifies the technical context and gaps addressed by SGMR-LPR.

### 2.1. Non-Semantic LPR Methods

Non-semantic LPR methods rely on geometric structures or 2D projections to construct place descriptors, emphasizing structural consistency and computational efficiency. They are divided into handcrafted feature-based and learning-based subcategories.

Handcrafted feature-based methods design manual rules to extract discriminative geometric features from 3D point clouds. A common paradigm is to convert unordered point clouds into structured representations (e.g., 2D projections, histograms, or grids) for feature encoding [11,12]. Representative works include polar coordinate transformation-based methods (Scan Context series [6,13] and LiDAR Iris [14]), multi-plane projection statistical methods (M2DP [15]), and voxel/grid histogram methods (NDT-histogram [16]). Additionally, segment-based methods (SegMatch [17]) and keypoint geometric consistency methods [18] also belong to this category. These methods have clear geometric interpretability but are sensitive to movable objects, viewpoint changes, and environmental variations due to over-reliance on fixed geometric patterns.

Learning-based methods leverage deep neural networks for automatic feature encoding, which can be further divided into 3D point/voxel-based and 2D projection-based approaches. 3D point/voxel-based methods directly process raw point clouds or voxelized data: PointNetVLAD [19] pioneered the use of PointNet [20] for local feature extraction and NetVLAD aggregation; subsequent works (PCAN [21], SOE-Net [22], DAGC [23], and PPT-Net [24]) introduced attention, graph convolution, or transformer to enhance local feature encoding. Voxel-based methods (MinkLoc3D [25] and SVT-Net [26]) reduce data sparsity while retaining structural information, but face computational efficiency challenges [27]. 2D projection-based methods convert point clouds into regular image structures (spherical, BEV, cylindrical, sinogram) [28,29,30,31,32,33], balancing efficiency and feature retention. BEV projection methods (BEVPlace [30,34]) are particularly mainstream due to rotation invariance and lightweight properties, which is why SGMR-LPR adopts the BEV input paradigm.

### 2.2. Semantic-Guided LPR Methods

Semantic information enhances LPR robustness by distinguishing geometrically similar but semantically different scenes. The existing methods are mainly divided into multi-stage (“segmentation-then-describing”) and end-to-end (“segmentation-while-describing”) frameworks.

Multi-stage methods rely on external semantic segmentation models to obtain semantic labels, which are then integrated into LPR via handcrafted structures. Typical approaches include semantic graph-based methods (SGPR [7]), semantic label-augmented descriptor methods (RINet [8] and SSC [6]), multi-level semantic aggregation methods (Locus [35]), and semantic consistency-constrained methods (SL_LPR [36]). These methods improve robustness to a certain extent but suffer from complex pipelines, error propagation between independent modules, and reliance on semantic ground truth during inference [10].

End-to-end methods address these limitations by introducing semantic segmentation auxiliary tasks to implicitly integrate semantic information into LPR. SG-LPR [10] proposed the “segmentation-while-describing” framework, where the auxiliary task only operates during training to guide the model to learn high-level implicit semantic features, enabling inference without external semantic predictions. Some works (e.g., [28]) have shown that augmenting input representations with semantic cues can improve recognition accuracy, but lack targeted integration of semantic auxiliary tasks and LPR objectives. Overall, end-to-end semantic-guided methods avoid multi-stage errors but fail to explicitly utilize semantic information for movable object suppression.

### 2.3. Movable Object Handling Methods

Movable or dynamic objects violate the static-world assumption and are widely recognized as a major source of failure in localization and loop closure detection. Extensive studies in SLAM demonstrate that dynamic objects introduce inconsistent geometric constraints between revisits, leading to false loop closures and degraded long-term localization performance [37,38]. To mitigate this issue, semantic-driven approaches leverage semantic segmentation to identify potentially movable categories (e.g., vehicles and pedestrians) and suppress their influence during loop closure or data association, typically by down-weighting or removing features belonging to dynamic regions [37,38]. Complementary geometry- or consistency-based methods detect dynamic points by exploiting spatial or temporal inconsistencies across scans, and filter them during scan matching or state estimation [39,40]. More recent dynamic-aware localization frameworks further argue that treating dynamic points as hard outliers is suboptimal, and instead model them as uncertain constraints integrated into the estimation process to improve robustness in highly dynamic environments [41]. Graph-Transformer [42] employs a two-stage, non-end-to-end framework for handling dynamic objects. In the first stage, a dynamic object segmentation module is used as a preprocessing step to remove dynamic points from the point clouds. This segmentation module is not integrated with the main model and is not jointly trained, making the entire framework dependent on the accuracy of the segmentation process. In the second stage, a scene graph is constructed using the remaining static points, and a transformer attention mechanism is applied to aggregate features and generate global descriptors. However, this approach relies on deterministic segmentation for “hard removal” of dynamic objects, which can limit its performance due to errors in the segmentation process. The inability to account for the inherent uncertainty of segmentation leads to potential loss of scene information or residual interference from incorrectly segmented dynamic objects.

In contrast, SGMR-LPR introduces a probabilistic, semantic-guided suppression mechanism that is tightly coupled with descriptor learning. By using soft semantic cues during feature encoding, SGMR-LPR handles movable object interference in an uncertainty-aware manner, making it more robust to dynamic environments and reducing the reliance on precise segmentation accuracy.

## 3. Methodology

### 3.1. Overview

SGMR-LPR is an end-to-end LPR framework designed to improve robustness to movable objects in dynamic outdoor environments. In large-scale driving scenarios, movable objects such as vehicles and pedestrians frequently introduce unstable structures into BEV representations, leading to significant appearance discrepancies across revisits. If not explicitly handled, these movable-object-induced variations can severely degrade the reliability of place recognition. To address this challenge, SGMR-LPR introduces semantic-guided mechanisms that explicitly identify and suppress the influence of movable objects during feature encoding while preserving structurally stable scene elements that are more reliable for place recognition.

**Figure 2:**
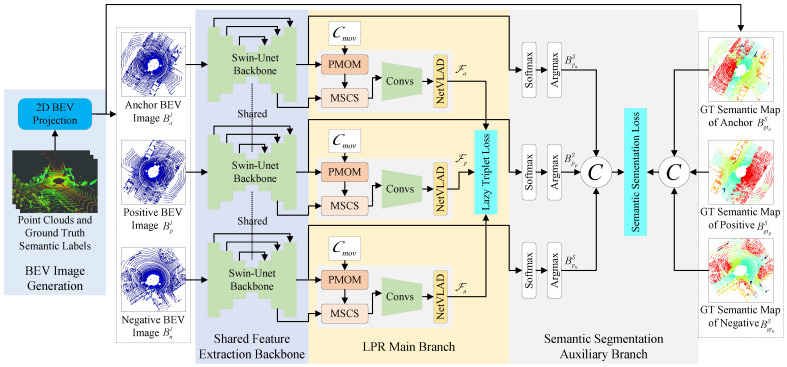
Overview of the proposed SGMR-LPR framework. The pipeline consists of three main parts: BEV image generation from raw LiDAR point clouds (gray area), a shared feature extraction backbone based on Swin-Unet [43] (blue area), and two task branches for LPR and auxiliary semantic segmentation (yellow area). The shared backbone outputs a high-level BEV feature tensor F and semantic logits Z, which are respectively used by the LPR main branch and the semantic segmentation auxiliary branch. The ground-truth semantic BEV is only used to supervise the segmentation branch during training.

As illustrated in Figure 2, the proposed framework consists of four main components: (1) BEV image generation from raw LiDAR point clouds; (2) a shared feature extraction backbone; (3) an LPR main branch equipped with explicit movable object suppression guided by semantic predictions; and (4) a semantic segmentation branch that provides dense semantic cues during training. Together, these components form a unified architecture that explicitly models movable object interference within an end-to-end trainable framework.

From an architectural perspective, SGMR-LPR adopts the “segmentation-while-describing” paradigm to inject semantic awareness into the feature learning process. Different from existing semantic-guided LPR methods where semantic supervision mainly acts as an implicit regularizer, SGMR-LPR establishes a direct functional connection between semantic predictions and the LPR task. Specifically, semantic outputs are explicitly transformed into probabilistic representations of movable objects, which are then used to guide movable-aware feature modulation in the LPR branch. Importantly, this design does not rely on external semantic models and does not introduce additional semantic annotations at inference time.

### 3.2. BEV Image Generation

**Figure 3:**
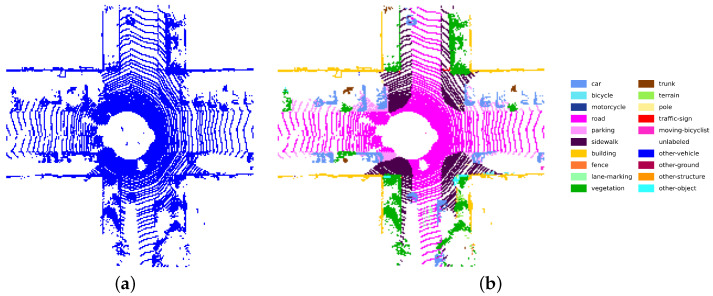
Samples of BEV generation: (**a**) displays the BEV image generated for 3D pointclouds based on point density, and (**b**) illustrates the ground-truth semantic map constructed from Semantic-KITTI [44].

Given a raw 3D LiDAR point cloud $P=\{p_{i}\left(x_{i},y_{i},z_{i}\right)\}$, we project it onto a 2D BEV plane to obtain a compact and structured representation suitable for convolutional and transformer-based networks, denoted as $B^{I}$, as illustrated in Figure 3a. Each point $p_{i}$ is mapped to pixel coordinates $\left(u_{i},v_{i}\right)$ in the BEV image according to

(1) $$\left\{\begin{array}{cc} u_{i} & =\frac{W}{2}+\left\lfloor \frac{x_{i}}{r}\right\rfloor ,\\ v_{i} & =\frac{H}{2}-\left\lfloor \frac{y_{i}}{r}\right\rfloor -1, \end{array}\right.$$

where *r* denotes the projection resolution, and (H,W) represents the spatial resolution of the BEV image. Each pixel value encodes the point density falling into the corresponding BEV grid cell.

For training the semantic segmentation auxiliary task, each BEV image is additionally associated with a semantic label map $B_{gt}^{S}$, as illustrated in Figure 3b, which is obtained by projecting point-wise semantic annotations from SemanticKITTI [44] onto the same BEV grid. These semantic labels are used only during training and are not required at inference time.

### 3.3. Shared Feature Extraction Backbone

SGMR-LPR employs a shared feature extraction backbone based on a Swin-Unet [43] architecture. The encoder utilizes window-based and shifted-window multi-head self-attention to capture long-range contextual dependencies with manageable computational cost [45], while the decoder progressively recovers spatial resolution through skip connections, preserving fine-grained geometric structures in BEV space.

Different from conventional designs that attach an independent semantic decoder, the backbone in SGMR-LPR is designed to jointly output high-level BEV features and dense semantic logits in a unified forward pass. Given an input BEV image $B^{I}$, the backbone produces

(2) $$\left(F,Z\right)=\mathrm{Backbone}\left(B^{I}\right),$$

where $F\in R^{H\times W\times D}$ denotes the high-level BEV feature map used for place recognition, and $Z\in R^{C\times H\times W}$ represents the semantic logits, with *C* being the number of semantic classes.

During training, Z is supervised by ground-truth semantic labels to provide dense semantic guidance for representation learning. During inference, semantic supervision is removed, and Z is used exclusively as an internal intermediate representation for probabilistic movable object mask generation. This design enables semantic-guided movable object suppression without introducing additional network components or external semantic models at test time.

### 3.4. LPR Main Branch

**Figure 4:**
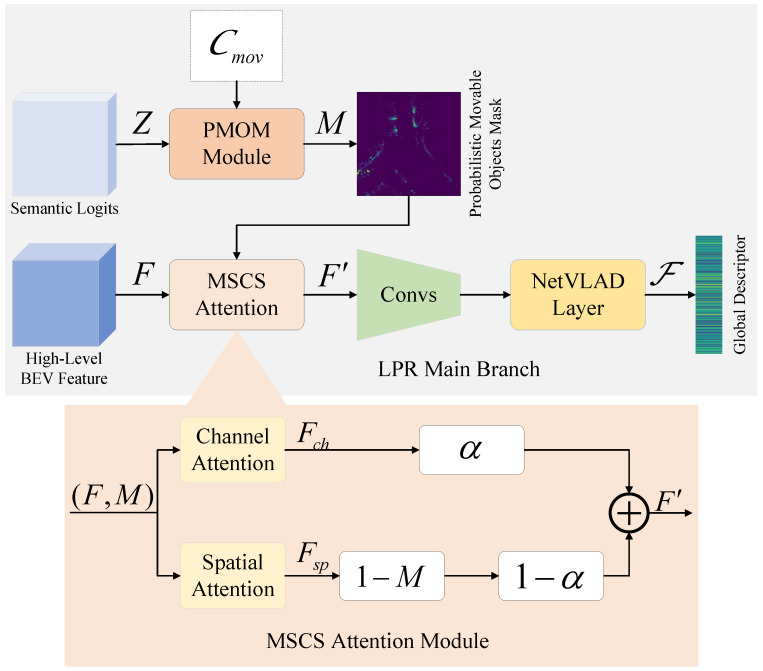
Architectures of the LPR main branch and the MSCS attention module.

The LPR main branch focuses on constructing a discriminative global place descriptor, where semantic-guided movable object suppression is embedded into the feature modulation process. The overall structure of this branch is illustrated in Figure 4.

#### 3.4.1. Probabilistic Movable Object Mask Generation

The shared backbone outputs semantic logits $Z\in R^{C\times H\times W}$, which serve as an intermediate representation and are converted into class-wise probability maps using a softmax operation along the channel dimension:

(3) P=Softmax(Z),

Let $C_{mov}$ denote the set of movable object categories (e.g., vehicles and pedestrians). The probabilistic movable object mask $M\in {\left[0,1\right]}^{H\times W}$ is defined as

(4) $$M\left(u,v\right)=\sum_{c\in C_{mov}}P_{c}\left(u,v\right),$$

which represents the likelihood that the BEV location (u,v) corresponds to a movable object.

This probabilistic formulation avoids hard binary decisions and naturally accounts for uncertainty in semantic predictions, enabling soft and confidence-aware suppression of movable regions during feature modulation.

#### 3.4.2. Movable-Suppressed Channel–Spatial Attention

To explicitly suppress the adverse influence of movable objects, we introduce a movable-suppressed channel–spatial (MSCS) attention mechanism. The objective of this module is to attenuate feature responses from regions with high movable-object likelihood while preserving structurally stable scene elements that are more reliable for place recognition.

Given the high-level BEV feature map F, channel attention and spatial attention are first computed independently following a CBAM-style [46] design. Channel attention emphasizes informative feature channels, whereas spatial attention highlights salient spatial regions in the BEV representation.

The probabilistic movable object mask M is then applied to the spatial attention pathway. Specifically, spatially attended features are modulated by (1−M), such that regions with high movable-object probability contribute less to subsequent feature encoding. The final modulated feature map $F^{\prime }$ is obtained as

(5) $$F^{\prime }=\alpha \;F_{ch}+\left(1-\alpha \right)\left(F_{sp}⊙\left(1-M\right)\right),$$

where $F_{ch}$ and $F_{sp}$ denote the channel-attended and spatial-attended features, respectively, ⊙ represents element-wise multiplication, and α∈[0,1] balances the two attention pathways. In our implementation, α is set to 0.5, a choice empirically motivated by the learnable-α convergence analysis presented in Section 4.5.4.

By embedding movable object suppression directly into the attention mechanism, MSCS enables soft attenuation of unstable regions while preserving spatial continuity and stable structural information, resulting in more robust BEV representations for place recognition in dynamic environments.

#### 3.4.3. Global Descriptor Aggregation

After movable-suppressed feature modulation by the MSCS module, the resulting feature map $F^{\prime }$ is first processed by three successive convolutional layers to adjust channel dimensions and spatial resolution, serving as a feature transformation stage before global aggregation. The transformed feature map is then aggregated by a NetVLAD layer [47] to produce a fixed-length global place descriptor:

(6) $$F=\mathrm{NetVLAD}(\mathrm{Convs}\left(F^{\prime }\right)),$$

where ${\mathrm{Conv}}_{3}\left(\cdot \right)$ denotes the three-layer convolutional transformation module. The resulting descriptor F is $\ell_{2}$-normalized and used for similarity computation in large-scale place retrieval.

### 3.5. Semantic Segmentation Auxiliary Branch

The semantic segmentation auxiliary branch in SGMR-LPR is intentionally lightweight and is designed to provide dense semantic guidance for movable-object modeling rather than acting as a standalone perception module. The shared backbone directly outputs semantic logits Z, which are supervised during training using ground-truth semantic maps $B_{gt}^{S}$. The predicted semantic map is obtained by

(7) $$B_{p}^{S}=\mathrm{Argmax}\left(\mathrm{Softmax}\left(Z\right)\right).$$

During training, this auxiliary supervision encourages the backbone to encode semantically informative representations that facilitate the identification of movable objects. During inference, no semantic labels are required and no explicit semantic predictions are used for downstream tasks. Instead, the semantic logits Z are retained internally and directly fed into the PMOM module to generate probabilistic movable-object masks for movable-aware feature modulation in the LPR main branch.

This minimalist design introduces no additional inference-time supervision or external semantic dependency, while maintaining a clear and functional semantic connection to movable object suppression.

### 3.6. Loss Function

Following SG-LPR, SGMR-LPR is optimized under a multi-task learning framework that jointly supervises place recognition and semantic segmentation. This design is intentionally retained to ensure fair comparison and stable optimization. Different from SG-LPR, where semantic supervision primarily serves as an auxiliary regularizer during training, in SGMR-LPR the learned semantic predictions are further exploited to construct probabilistic movable object masks and semantic-aware attention, which implicitly modulate feature aggregation for robust place recognition. The overall training loss is defined as

(8) $$L=\lambda_{lt}L_{lt}+\lambda_{seg}L_{seg},$$

where $L_{lt}$ denotes the place recognition loss, $L_{seg}$ denotes the semantic segmentation loss, and $\lambda_{lt}$ and $\lambda_{seg}$ balance the two objectives during training.

The two task-level weights are set equally to $\lambda_{lt}=\lambda_{seg}=1.0$, reflecting the equal importance assigned to the place recognition task and the semantic segmentation auxiliary task in our multi-task learning design. This prevents either objective from dominating the gradients during the early stages of training. The internal weights of the segmentation loss, $\lambda_{ce}$ and $\lambda_{dice}$, are set to 0.4 and 0.6 respectively, following the original configuration of Swin-Unet [43] and its subsequent adoption in SG-LPR [10] for the same semantic segmentation auxiliary task. This configuration has been validated to effectively mitigate class imbalance and background dominance in BEV representations.

#### 3.6.1. Place Recognition Loss

For the place recognition task, we adopt the lazy triplet loss [19], a widely used metric learning objective for large-scale place retrieval:

(9) $$L_{lt}=\max\left(0,m+\delta_{p}-\min_{i}\delta_{n_{i}}\right),$$

where $\delta_{p}$ denotes the distance between the anchor descriptor and its corresponding positive descriptor, $\delta_{n_{i}}$ denotes the distance to the *i*-th negative descriptor, and *m* is a predefined margin. By focusing on the hardest negative sample within each training tuple, this loss encourages discriminative global descriptors while maintaining training efficiency.

#### 3.6.2. Semantic Segmentation Loss

The semantic segmentation objective provides dense supervision to guide the shared backbone toward learning semantically meaningful representations. In contrast to methods that directly use semantic predictions for post-processing or hard filtering, SGMR-LPR leverages the segmentation outputs to generate probabilistic movable object masks and semantic-aware attention weights, enabling uncertainty-aware feature modulation.

The segmentation loss is formulated as a weighted combination of cross-entropy loss and Dice loss:

(10) $$L_{seg}=\lambda_{ce}L_{ce}+\lambda_{dice}L_{dice},$$

where $\lambda_{ce}$ and $\lambda_{dice}$ control the relative contributions of each term.

The cross-entropy loss is defined as

(11) $$L_{ce}\left(Y,\hat{Y}\right)=-\frac{1}{N}\sum_{i=1}^{N}\sum_{c=1}^{C}y_{i}^{c}\cdot log({\hat{y}}_{i}^{c}),$$

where *Y* and $\hat{Y}$ denote the predicted semantic probability map and the corresponding ground-truth label map, respectively. Here, *N* is the number of pixels in a BEV image, *C* is the number of semantic classes, $y_{i}^{c}$ represents the predicted probability of pixel *i* belonging to class *c*, and ${\hat{y}}_{i}^{c}$ denotes the ground-truth label.

To mitigate class imbalance and background dominance in BEV representations, we further incorporate the Dice loss [43]:

(12) $$L_{dice}\left(Y,\hat{Y}\right)=\frac{1}{C}\sum_{c=1}^{C}w_{c}\left(1-\frac{2\sum_{i=1}^{N}\sum_{j=1}^{N}\left(y_{ij}^{c}\times {\hat{y}}_{ij}^{c}\right)+\epsilon }{\sum_{i=1}^{N}\sum_{j=1}^{N}{\left(y_{ij}^{c}\right)}^{2}+\sum_{i=1}^{N}\sum_{j=1}^{N}{\left({\hat{y}}_{ij}^{c}\right)}^{2}+\epsilon }\right),$$

where $y_{ij}^{c}$ and ${\hat{y}}_{ij}^{c}$ denote the predicted and ground-truth labels at pixel location (i,j) for class *c*, respectively. The class weight $w_{c}$ is set to 1.0 for all the classes, and ϵ is a smoothing term set to $10^{-5}$.

### 3.7. Algorithm Summary

To provide a clearer understanding of the novelty and operational flow of the proposed framework, the end-to-end forward pass and joint training procedures of SGMR-LPR are summarized in Algorithm 1. The algorithm explicitly highlights our core contributions: the uncertainty-aware probabilistic masking and the movable-suppressed feature modulation.
**Algorithm 1** SGMR-LPR: Forward Pass and Joint Training Procedure   **Input:** Raw LiDAR point cloud *P*; Ground-truth semantic BEV $B_{gt}^{S}$ (if training phase)   **Parameters:** Movable class set $C_{mov}$; Attention balance coefficient α=0.5; Loss weights $\lambda_{lt}=\lambda_{seg}=1.0$, $\lambda_{ce}=0.4$, $\lambda_{dice}=0.6$   **Output:** $\ell_{2}$-normalized global place descriptor F   ***Stage 1: Unified Representation Learning***
  1:Generate BEV image $B^{I}$ from raw point cloud *P*                                          ▹Equation (1)  2:$F,Z\leftarrow \mathrm{Backbone}(B^{I})$                                        ▹F: high-level BEV features, Z: semantic logits***Stage 2: Novel Semantic-Guided Feature Modulation (Ours)****— Probabilistic Movable Object Masking (PMOM) —*  3:P←Softmax(Z,dim=channel)  4:$M\leftarrow \mathrm{Clamp}(\sum_{c\in C_{mov}}P_{c},\;0,\;1)$                                     ▹ Soft uncertainty-aware masking, Equation (4)*— Movable-suppressed Channel-Spatial Attention (MSCS) —*  5:$F_{ch}\leftarrow \mathrm{ChannelAttention}\left(F\right)$  6:$F_{sp}\leftarrow \mathrm{SpatialAttention}\left(F\right)$  7:$F^{\prime }\leftarrow \alpha F_{ch}+\left(1-\alpha \right)\left(F_{sp}⊙\left(1-M\right)\right)$                                       ▹ Explicit dynamic suppression, Equation (5)***Stage 3: Global Descriptor Aggregation***  8:$F_{conv}\leftarrow \mathrm{Conv}\_\mathrm{Block}\left(F^{\prime }\right)$                                          ▹ Spatial & channel dimension adjustment  9:$F\leftarrow L2\_\mathrm{Normalize}(\mathrm{NetVLAD}\left(F_{conv}\right))$***Stage 4: Multi-Task Joint Optimization (Training phase only)***10:**if** training **then**11:                                               ▹ Given descriptors $F_{a},F_{p},\left\{F_{n_{i}}\right\}$ extracted from a training tuple12:      $L_{lt}\leftarrow \mathrm{LazyTripletLoss}\left(F_{a},F_{p},\left\{F_{n_{i}}\right\}\right)$13:      $L_{seg}\leftarrow \lambda_{ce}L_{ce}\left(P,B_{gt}^{S}\right)+\lambda_{dice}L_{dice}\left(P,B_{gt}^{S}\right)$                                            ▹ Equations (10)–(12)14:      $L\leftarrow \lambda_{lt}L_{lt}+\lambda_{seg}L_{seg}$                                                        ▹ Equation (8)15:      Update model parameters by minimizing L16:**end if**17:**return** F

## 4. Results

### 4.1. Datasets and Experimental Settings

#### 4.1.1. Datasets

We evaluate SGMR-LPR on three public LiDAR datasets and one self-collected dataset, covering standard urban driving scenarios, highly dynamic traffic environments, long-term campus scenes, and sparse off-road terrains. This evaluation protocol is designed to assess conventional place recognition performance, robustness under dynamic scenes, and cross-domain generalization across distinct environment types.

**KITTI [48].** The KITTI dataset provides large-scale urban driving LiDAR scans collected using a Velodyne HDL-64E sensor, along with accurate ground-truth poses. In this work, sequences 00, 02, and 08 are used for model training and standard place recognition evaluation, following widely adopted LPR protocols. These sequences contain sufficient loop closures and serve as the primary benchmark for evaluating conventional LPR performance.

**KITTI-360 [49].** KITTI-360 extends KITTI with denser annotations and more complex urban scenes, featuring frequent appearances of vehicles, pedestrians, and other movable objects. To specifically evaluate robustness under dynamic environments, we conduct experiments on sequences 0000, 0002, 0004, 0005, 0006, and 0009.

**NCLT [50].** The NCLT dataset consists of long-term LiDAR scans collected on a university campus using a Velodyne HDL-32 sensor over a period of more than one year. It captures substantial variations in seasons, weather conditions, illumination, and scene appearance. To evaluate long-term generalization, sequence “2012-01-15” is used to construct the database, while the remaining sequences (“2012-02-04”, “2012-03-17”, “2012-06-15”, “2012-09-28”, “2012-11-16”, and “2013-02-23”) are used as query sequences.

**OffRoad-LPR [51].** OffRoad-LPR is a self-collected LiDAR dataset captured in large-scale off-road environments, characterized by unstructured terrains and sparse geometric features with limited dynamic objects. It is used to evaluate cross-domain generalization of LPR models under structurally distinct and feature-sparse conditions. The dataset contains two sequences, with sequence 00 used as the database and sequence 01 as the query set. As a private dataset, OffRoad-LPR is used only for evaluation and not for training or parameter tuning.

#### 4.1.2. Experimental Settings

The proposed model is implemented using the PyTorch-1.10 framework and trained on a single NVIDIA A100 GPU with 40 GB of memory. The training follows the “segmentation-while-describing” paradigm, where semantic annotations are used only to supervise the auxiliary semantic segmentation task during training and are not required at inference time.

**BEV representation.** Each 3D point cloud is projected onto a BEV plane with a grid resolution of r=0.2 m and a maximum projection radius of 11.2 m, resulting in BEV images of size 224×224.

**Training strategy.** SGMR-LPR is trained exclusively on the KITTI dataset using a triplet-based learning scheme. For each training tuple, one positive sample ($k_{p}=1$) and ten negative samples ($k_{n}=10$) are selected for each anchor. Two point clouds are considered a positive pair if their ground-truth pose distance is less than 3 m, and a negative pair if the distance exceeds 20 m. Following SGPR [7], a leave-one-out cross-validation protocol is adopted on KITTI, where sequences 00, 02, and 08 are alternately used for testing and the remaining sequences for training.

**Optimization.** The network is trained from scratch using the Adam optimizer with an initial learning rate of $1\times 10^{-5}$ and an exponential learning rate scheduler. Random rotations around the vertical axis are applied as data augmentation.

**Loss configuration.** The weighting coefficients are set to $\lambda_{lt}=1.0$ and $\lambda_{seg}=1.0$. Within the segmentation loss, the weights for the cross-entropy and Dice terms are set to $\lambda_{ce}=0.4$ and $\lambda_{dice}=0.6$, respectively.

The rationale for these choices is detailed in Section 3.6, and the configuration follows the settings validated in Swin-Unet [43] and SG-LPR [10].

**Movable object class set.** The movable object category set $C_{mov}$ is used to generate probabilistic movable object masks from the predicted semantic logits. In both training and inference, $C_{mov}$ is defined as

(13) $$C_{mov}=\left\{1,2,3,4,5,6,7,8,9,10,25,26,27,28,29,30,31,32\right\},$$

which correspond to the following movable semantic classes after label remapping in the SemanticKITTI [44] dataset: {car, bicycle, bus, motorcycle, on-rails, truck, other-vehicle, person, bicyclist, motorcyclist, moving-car, moving-bicyclist, moving-person, moving-motorcyclist, moving-on-rails, moving-bus, moving-truck, moving-other-vehicle}. This set covers all the movable object categories provided by SemanticKITTI under our task-specific label reorganization.

**Model configuration.** The shared backbone is configured following the Swin-Unet architecture described in [43]. The NetVLAD aggregation layer uses 64 clusters and produces a 256-dimensional global descriptor.

#### 4.1.3. Evaluation Metrics

To evaluate the performance of SGMR-LPR, we adopt both quantitative and qualitative evaluation metrics. Quantitatively, the maximum $F_{1}$ score and Recall@1/*N* are used to measure place recognition accuracy and retrieval performance. Precision (*P*), recall (*R*), and the $F_{1}$ score are defined as

(14) $$\left\{\begin{array}{cc} P & =\frac{TP}{TP+FP},\\ R & =\frac{TP}{TP+FN},\\ F_{1} & =2\cdot \frac{P\cdot R}{P+R}, \end{array}\right.$$

where TP, FP, and FN denote the numbers of true positives, false positives, and false negatives, respectively. The maximum $F_{1}$ score is obtained by sweeping the similarity threshold.

Qualitatively, precision–recall (PR) curves, Top-1 retrieval results along the trajectory, and feature map visualizations are reported to provide intuitive analysis of retrieval behavior and feature responses under different environmental conditions.

### 4.2. Comparison with State-of-the-Art

We evaluate SGMR-LPR against a broad range of representative LPR methods, including handcrafted approaches (M2DP [15], Scan Context (SC) [13], and LiDAR Iris (LI) [14]), learning-based methods (PointNetVLAD (PNV) [19], DiSCO [31], BEVPlace [30], and BEVPlace++ [34]), and semantic-aware approaches (SSC [6], SGPR [7], Locus [35], RINet [8], SC_LPR [36], and SG-LPR [10]). All the models are evaluated following the standard protocols reported in [8,10]. SGMR-LPR is trained only on the KITTI dataset.

**Quantitative Results on KITTI.** Table 1 reports the maximum $F_{1}$ scores on sequences 00, 02, and 08 of the KITTI dataset, together with their mean performance. These sequences present different levels of difficulty: sequence 02 contains multiple geometrically similar repetitive scenes in addition to reverse loop closures, which places high demands on feature discriminability, while sequence 08 includes pronounced reverse loop closures, enabling evaluation under extreme viewpoint variations.

As shown in the table, SGMR-LPR consistently achieves the best or second-best performance across all the evaluated sequences and attains the highest average $F_{1}$ score among all the compared methods. In particular, SGMR-LPR outperforms the strongest competitors on the more challenging sequences 02 and 08. Compared with SG-LPR, the proposed method yields consistent improvements on all three sequences.

**Robustness to Viewpoint Variations.** To further assess robustness to viewpoint changes, we evaluate all the methods on the randomly rotated KITTI dataset, constructed by applying random rotations around the *z*-axis to the original point clouds. This setting follows common practice in LPR and simulates viewpoint variations encountered in real-world scenarios [7,8].

As reported in Table 1, SGMR-LPR maintains consistently high performance under random rotations, with only a marginal performance gap between the raw and rotated datasets, and the Diff value for SGMR-LPR is −0.003.

**Figure 5:**
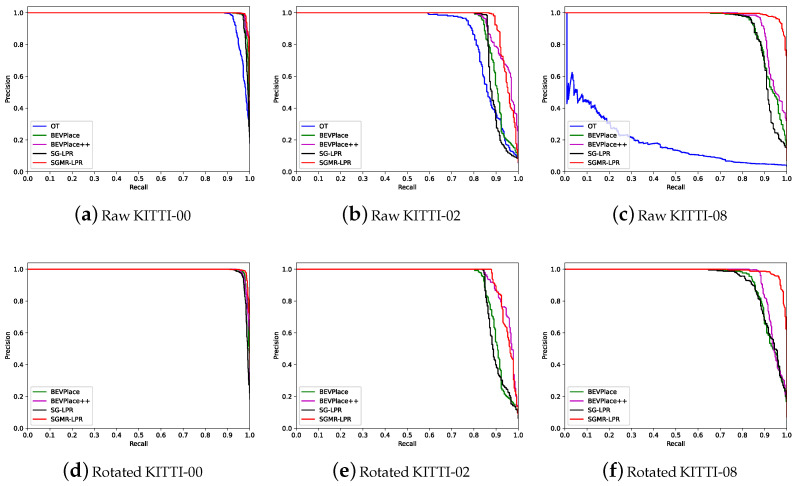
The precision–recall curves on multiple sequences of KITTI dataset.

**PR Curves Comparison.** Figure 5 presents the precision–recall (PR) curves of different methods on the raw KITTI and randomly rotated KITTI datasets, covering sequences 00, 02, and 08.

On the raw KITTI dataset, SGMR-LPR consistently achieves the largest PR curve coverage across all the sequences. On sequence 00, except for OverlapTransformer (OT) [29], the compared methods exhibit similar PR behaviors, with SGMR-LPR maintaining a slight advantage, particularly at high recall levels (as illustrated in Figure 5a). On the more challenging sequence 02, the PR curves begin to diverge: although SGMR-LPR preserves the highest overall coverage, its precision decreases marginally faster than BEVPlace++ when the recall exceeds approximately 0.93 (as illustrated in Figure 5b). On sequence 08, which involves pronounced reverse loop closures and extreme viewpoint variations, SGMR-LPR clearly outperforms all the competing methods in terms of both curve coverage and precision decay behavior.

Similar trends are observed on the randomly rotated KITTI dataset. On rotated sequence 00, all the methods yield nearly overlapping PR curves, while SGMR-LPR retains slightly better coverage and slower precision degradation (as shown in Figure 5d). On rotated sequence 02, SGMR-LPR and BEVPlace++ show comparable performance, with SGMR-LPR being more stable at lower recall and BEVPlace++ exhibiting a mild advantage in the mid-recall range, after which the two curves nearly coincide (as shown in Figure 5e). On rotated sequence 08, SGMR-LPR again demonstrates the strongest overall performance (as shown in Figure 5f).

**Figure 6:**
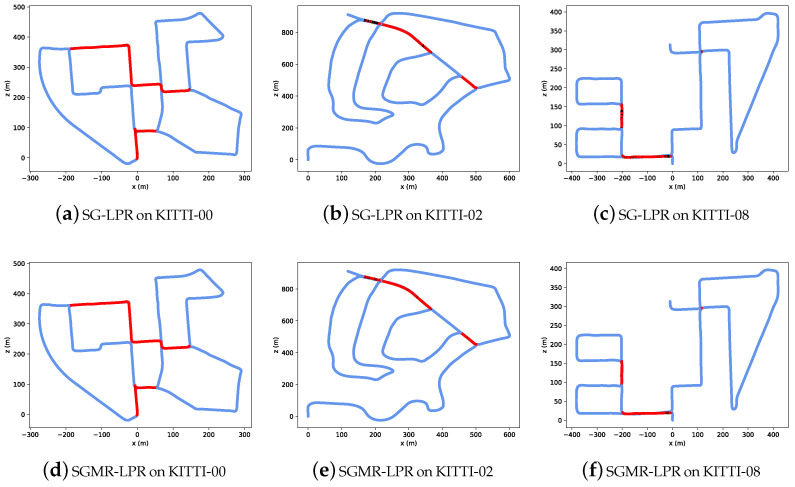
Qualitative performance at top-1 retrieval of SG-LPR and SGMR-LPR on multiple KITTI sequences along the trajectory. Red: true positives, black: false negatives, blue: true negatives, cyan: false positives (no such cases are observed in this figure).

**Top-1 Retrieval Results Comparison.** Figure 6 visualizes the Top-1 retrieval results along the trajectories of KITTI sequences 00, 02, and 08 for SG-LPR and SGMR-LPR. Each point on the trajectory is color-coded to indicate the retrieval outcome, enabling an intuitive comparison of false negatives across different scene dynamics.

As illustrated in Figure 6a,d, on sequence 00, both SG-LPR and SGMR-LPR exhibit very few false negatives. In contrast, clear performance differences emerge on the more challenging sequences 02 and 08. As illustrated in Figure 6b,e, on sequence 02, which contains reverse loop closures as well as multiple geometrically similar repetitive scenes, SG-LPR produces a noticeably higher number of false negatives, whereas SGMR-LPR maintains substantially more correct Top-1 matches along the trajectory. A similar trend is observed on sequence 08, where frequent reverse loop closures lead to pronounced viewpoint changes: SGMR-LPR consistently reduces false negatives compared with SG-LPR, as illustrated in Figure 6c,f. Notably, no false positive cases are observed for either model across all the sequences.

### 4.3. Robustness in Highly Dynamic Scenes

**Quantitative Results on KITTI-360.** We further evaluate the robustness of SGMR-LPR in highly dynamic urban environments on the KITTI-360 dataset, which features dense traffic participants and frequent scene changes. As reported in Table 2, SGMR-LPR achieves the best performance on all six evaluated sequences. Further more, SGMR-LPR attains the highest mean maximum $F_{1}$ score of 0.978. Several competitive semantic-aware or learning-based methods (e.g., SSC, RINet, and SG-LPR) achieve strong results on individual sequences. Compared with the semantic-guided baseline SG-LPR, SGMR-LPR yields higher $F_{1}$ scores on every evaluated sequence.

**Figure 7:**
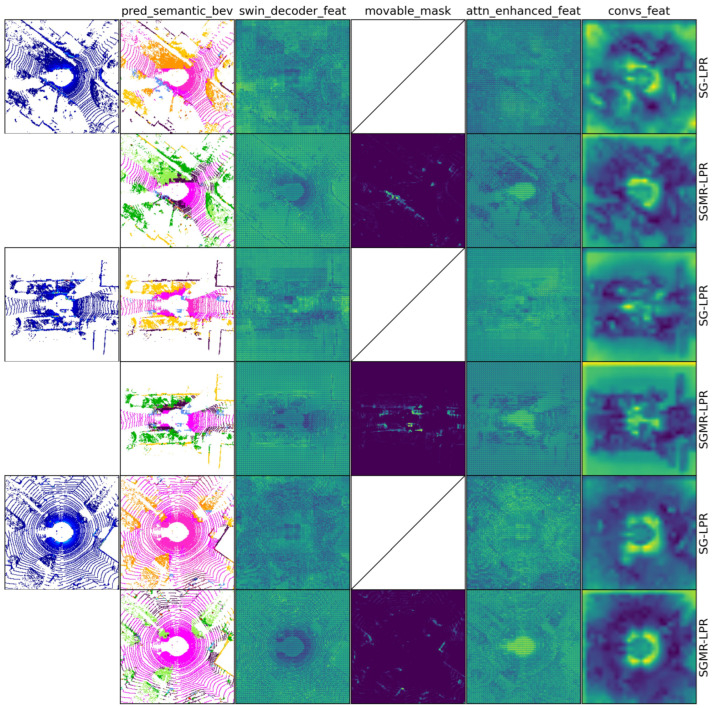
Qualitative comparison of SG-LPR and SGMR-LPR on the KITTI-360 dataset. The first column shows selected raw BEV inputs, while columns 2–6 visualize intermediate outputs, including the predicted semantic BEV, Swin decoder features, movable masks, attention-enhanced features, and convolution-adjusted features (as labeled). For each sample, odd rows correspond to SG-LPR and even rows to SGMR-LPR. The movable mask column is absent for SG-LPR, as it does not employ movable object masking. Compared with SG-LPR, SGMR-LPR exhibits clearer suppression of dynamic regions in attention-enhanced and convolution-adjusted features, leading to more stable high-level representations.

**Qualitative Results.** To further analyze the behavior of SGMR-LPR in highly dynamic environments, we present qualitative comparisons with SG-LPR on the KITTI-360 dataset in Figure 7. Since KITTI-360 does not provide frame-wise semantic ground-truth annotations, and both models are trained on the KITTI dataset, the predicted semantic BEV maps may contain uncertainties. Our analysis focuses on how the predicted semantic cues are exploited to modulate intermediate feature representations.

As shown in the figure, SG-LPR does not employ movable object masking, and the corresponding positions in the movable mask column are intentionally left blank and filled with diagonal lines. The attention-enhanced features of SG-LPR are obtained using a standard CBAM [46] module applied to the Swin decoder features, whereas SGMR-LPR employs the proposed MSCS attention mechanism, which explicitly integrates movable masks into the attention modulation process.

Despite potential inaccuracies in the predicted semantic BEV maps, SGMR-LPR consistently suppresses feature responses in regions indicated as movable by the generated masks. This suppression is reflected in both the attention-enhanced features and the subsequent convolution-adjusted features, where dynamic-object-dominated regions exhibit lower activations and improved spatial coherence. In contrast, SG-LPR retains relatively strong responses in these regions.

### 4.4. Generalization Performance

We evaluate the cross-domain generalization ability of SGMR-LPR on the NCLT and OffRoad-LPR datasets without any fine-tuning or domain-specific adaptation. All the models are trained on the KITTI dataset using its semantic annotations, while during testing on NCLT and OffRoad-LPR, no semantic ground-truth or external semantic information is available.

**Results on NCLT.** The NCLT dataset is collected using a 32-beam LiDAR sensor, resulting in significantly sparser point clouds and BEV representations compared to KITTI. As reported in Table 3, SGMR-LPR achieves the highest mean Recall@1 score among all the compared methods.

Specifically, SGMR-LPR consistently ranks among the top-performing methods across all six test sequences and achieves the best results on four of them. BEVPlace++ attains strong performance on earlier sequences and SG-LPR remains competitive overall.

**Figure 8:**
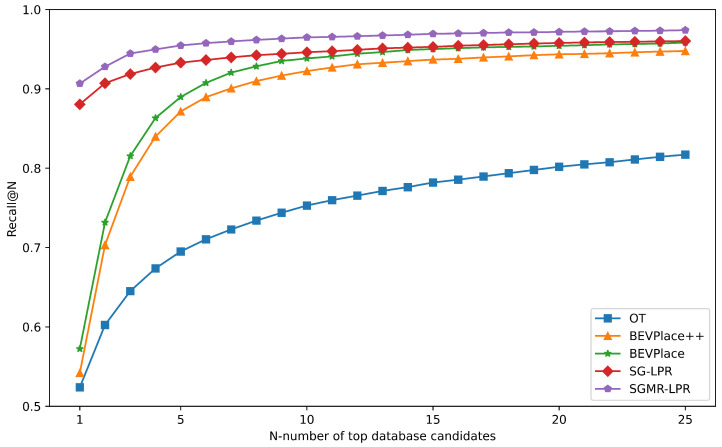
Recall@N of different models on OffRoad-LPR dataset.

**Results on OffRoad-LPR.** To further examine generalization to structurally distinct environments, we evaluate SGMR-LPR on the OffRoad-LPR dataset, which consists of unstructured off-road scenes with sparse geometry. Figure 8 reports the Recall@N curves of representative baseline methods.

As shown in the figure, SGMR-LPR consistently outperforms all the compared approaches across the entire Recall@N range. Its Recall@1 reaches 0.9, and the Recall@N curve fully envelops those of other methods.

### 4.5. Ablation Study

#### 4.5.1. Effectiveness of Explicit Movable Object Suppression

To evaluate the effectiveness of explicit movable object modeling and suppression, we compare SGMR-LPR with its baseline SG-LPR on multiple KITTI sequences. Table 4 reports the maximum $F_{1}$ scores of SG-LPR and SGMR-LPR on KITTI sequences 00, 02, and 08. SGMR-LPR achieves higher scores than SG-LPR on all the sequences, with mean scores of 0.962 and 0.932, respectively.

#### 4.5.2. Ablation on Masking Strategy for Movable Object Suppression

Table 5 compares three masking variants: no masking (SGMR-LPR-MN), hard masking (SGMR-LPR-MH), and soft masking (SGMR-LPR). Soft masking achieves the highest scores on all the sequences, with mean $F_{1}$ of 0.962, compared to 0.934 for no masking and 0.939 for hard masking. The gains are most pronounced on sequences 02 and 08.

**Figure 9:**
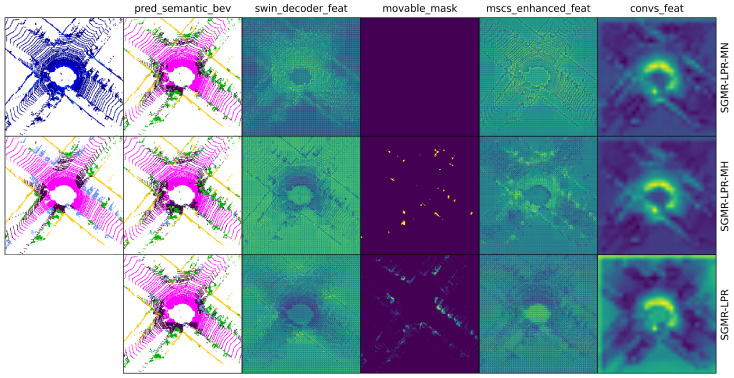
Qualitative visualization of different masking strategies in SGMR-LPR. The first column shows the raw BEV input and the ground-truth semantic BEV for reference. Rows from top to bottom correspond to SGMR-LPR-MN (no masking), SGMR-LPR-MH (hard masking), and the full SGMR-LPR (soft masking). Compared with no masking and hard masking, the proposed soft masking strategy produces more coherent movable object suppression and cleaner high-level feature representations in dynamic regions.

Figure 9 visualizes the intermediate features for the three strategies. Soft masking produces more coherent movable-object masks and cleaner high-level feature representations in dynamic regions compared to no masking and hard masking.

#### 4.5.3. Ablation on Attention Mechanism Design

Table 6 compares the standard CBAM attention with the proposed MSCS attention. MSCS outperforms CBAM on all the sequences, achieving mean $F_{1}$ of 0.962 versus 0.927. On sequence 08, MSCS yields a relative improvement of 6.9% over CBAM.

#### 4.5.4. Ablation on Attention Coefficient Strategy

**Figure 10:**
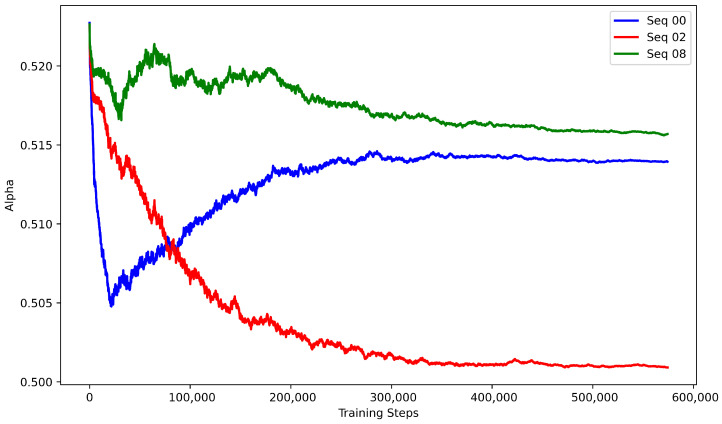
Variation of alpha during training on KITTI dataset.

To investigate the impact of the balance coefficient α between channel and spatial attention in the MSCS module, we compare a fixed strategy (α=0.5) with a learnable strategy. For the learnable strategy, we adopt a leave-one-sequence-out cross-validation procedure on the KITTI dataset (sequences 00, 02, and 08): each time, one sequence serves as the validation set while the other two are used for training, and α is initialized randomly. Figure 10 shows the training dynamics of α under this protocol. When sequences 00, 02, and 08 are used as the validation set in turn, α converges to 0.515, 0.515, and 0.500, respectively. This consistent convergence towards approximately 0.5 indicates that a balanced trade-off between channel and spatial attention emerges naturally from the data. These results provide the empirical justification for the fixed setting of α=0.5 adopted in the MSCS module (Section 3.4.2).

Table 7 compares the two strategies in terms of maximum $F_{1}$ scores. The fixed strategy consistently outperforms the learnable variant across all three sequences, achieving a mean $F_{1}$ score of 0.962 compared to 0.927 for the learnable strategy. Notably, although the learnable α converges to values near 0.5, its performance is inferior to directly fixing α=0.5. A possible explanation is the additional optimization difficulty and potential training instability introduced by treating α as a free parameter. Therefore, based on the convergent behavior and performance comparison, we adopt the fixed strategy (α=0.5) in SGMR-LPR, which strikes an effective balance between accuracy and training stability.

## 5. Discussion

This section interprets the experimental results in the context of prior work and discusses the implications and limitations of the proposed approach.

### 5.1. Discussion of Comparison with State-of-the-Art

As shown in Table 1, the superior performance of SGMR-LPR on KITTI sequences, particularly the consistent improvements over SG-LPR on all three sequences, demonstrates that explicitly suppressing movable-object interference during feature encoding effectively enhances place recognition while preserving strong baseline representation capability. The strong results on the more challenging sequences 02 and 08 further indicate its superior robustness in complex and dynamic environments. The marginal performance gap between raw and randomly rotated datasets (Diff = −0.003) suggests that the proposed movable-aware suppression mechanism does not introduce sensitivity to rotational perturbations, thereby preserving stable and discriminative place representations under viewpoint variations.

The PR curves (as illustrated in Figure 5) corroborates the quantitative findings. The largest curve coverage achieved by SGMR-LPR across all the sequences, especially the clear advantage on sequence 08 under extreme viewpoint changes, highlights its effectiveness in handling challenging scenarios. The slight precision decay relative to BEVPlace++ on sequence 02 at very high recall levels does not undermine its overall superior coverage, and on rotated datasets the behavior remains consistent, confirming robustness to rotations.

The Top-1 retrieval visualized in Figure 6 further support these conclusions. The reduction in false negatives on sequences 02 and 08, without any increase in false positives, indicates that the improvements achieved by SGMR-LPR mainly stem from enhanced recall rather than increased ambiguity. The absence of false positives across all the sequences suggests that the method maintains high precision while improving recall. Overall, both the quantitative and qualitative results consistently demonstrate the robustness and effectiveness of SGMR-LPR in comparison to state-of-the-art methods.

### 5.2. Discussion of Robustness in Highly Dynamic Scenes

The superior performance of SGMR-LPR on KITTI-360, where it achieves the highest mean $F_{1}$ score and outperforms all the competing methods on every sequence (as shown in Table 2), demonstrates its enhanced robustness in highly dynamic urban environments with dense traffic participants and frequent scene changes. While several methods achieve strong results on individual sequences, none maintain consistent superiority across all the splits, whereas SGMR-LPR exhibits stable and uniformly high performance. The consistent improvements over SG-LPR on every evaluated sequence highlight the benefit of explicitly suppressing movable object interference during feature encoding, suggesting that the proposed movable-suppressed attention mechanism provides complementary robustness beyond semantic-aware place representations, particularly in large-scale urban environments with frequent dynamic object interactions.

The qualitative results provides intuitive evidence supporting these quantitative findings (as illustrated in Figure 7). Despite uncertainties in the predicted semantic maps due to domain shift from KITTI to KITTI-360, SGMR-LPR effectively leverages semantic cues to guide feature suppression in a soft and robust manner. The clearer suppression of dynamic regions in attention-enhanced and convolution-adjusted features, compared to SG-LPR which retains stronger responses in these regions, indicates that the MSCS attention mechanism contributes to more stable high-level representations. This behavior confirms that SGMR-LPR does not rely on perfectly accurate semantic predictions, aligning with the quantitative improvements reported in Table 2.

### 5.3. Discussion of Generalization Performance

The results on NCLT (as shown in Table 3) demonstrate strong generalization capability of SGMR-LPR under sparse input conditions. Despite the significant reduction in sensing density from 64-beam to 32-beam LiDAR, which poses additional challenges for feature extraction and place recognition, SGMR-LPR achieves the highest mean Recall@1 among all the methods. While BEVPlace++ shows strong performance on earlier sequences and SG-LPR remains competitive overall, SGMR-LPR exhibits more stable performance across different temporal splits, leading to the highest average Recall@1. This indicates that the representations learned with semantic-guided movable suppression on KITTI generalize well to sparser LiDAR configurations and different environments.

The results on OffRoad-LPR (as illustrated in Figure 8) further confirm generalization to structurally distinct environments. SGMR-LPR consistently outperforms all the compared approaches across the entire Recall@N range in unstructured off-road scenes with sparse geometry. The Recall@1 reaching 0.9 and the curve fully enveloping those of other methods indicate robust retrieval performance in sparse and irregular environments. This suggests that the proposed movable-suppressed attention mechanism effectively enhances the stability of place representations under significant structural variations.

Taken together with the results on NCLT, these findings demonstrate that SGMR-LPR generalizes well across different LiDAR configurations, scene structures, and sensing conditions, maintaining reliable place recognition performance beyond the urban driving scenarios observed during training. The strong performance in both sparser urban settings and structurally distinct off-road environments confirms the robustness and transferability of the learned representations.

### 5.4. Discussion of Ablation Studies

The ablation studies validate the core design choices of SGMR-LPR. Explicit movable object suppression via PMOM consistently improves performance over SG-LPR, confirming that directly modulating features based on movable-object awareness enhances robustness in dynamic environments. Among masking strategies, soft masking outperforms both no masking and hard masking, with the largest gains on challenging sequences; this indicates that probabilistic attenuation better preserves structural context while suppressing dynamic interference, leading to more discriminative descriptors. The MSCS attention mechanism proves more effective than standard CBAM, particularly under extreme viewpoint variations (sequence 08), demonstrating the benefit of integrating movable-aware cues into joint spatial-channel attention. Finally, the fixed attention coefficient (α=0.5) outperforms a learnable counterpart despite the latter converging to similar values, suggesting that additional parametric flexibility is unnecessary and a fixed balance provides optimal stability and performance. Collectively, these findings establish the efficacy of the proposed movable-suppressed feature modulation paradigm.

### 5.5. Discussion of Computational Efficiency

The evaluation of computational efficiency is conducted on the OffRoad-LPR [51] dataset, which comprises sequences 00 and 01 with a total length of 17.5 km. The measurements are performed on a system equipped with an NVIDIA RTX 3070 GPU with 8 GB of VRAM, and the results are summarized in Table 8.

As shown in the table, the efficiency characteristics of each method are closely related to their architectural designs. OT [29] employs a conventional transformer based on multi-head self-attention, which leads to a relatively large parameter count; however, since it processes single-channel range images projected from 3D LiDAR point clouds, it achieves low computational cost and high inference speed. BEVPlace [30] constructs its model using group convolutions but requires multiple data augmentation operations on the input BEV images during computation; consequently, despite its small parameter scale, its computational cost is relatively high. BEVPlace++ [34] adopts a convolution-based architecture and achieves the smallest parameter count, yet it also requires multiple random rotations of the input BEV images with separate feature extraction for each rotation, resulting in high computational cost. Compared with SGMR-LPR, SG-LPR has no explicit interaction between the semantic segmentation auxiliary branch and the LPR main branch; nevertheless, it employs a CBAM [46] attention module in the LPR main branch, which leads to a higher computational cost. Overall, although SGMR-LPR does not achieve the optimal parameter scale or computational cost among the compared methods, its inference efficiency remains sufficient to meet real-time requirements.

## 6. Conclusions

In this paper, we presented SGMR-LPR, a semantic-guided LiDAR-based place recognition framework that explicitly mitigates the adverse impact of movable objects in dynamic environments. By coupling place recognition with an auxiliary semantic segmentation task during training, SGMR-LPR effectively exploits semantic cues to enable movable-aware feature modulation while avoiding additional semantic dependencies at inference time. This design allows the network to focus on stable structural information and suppress unreliable features induced by movable objects, resulting in more robust and discriminative global place descriptors. The proposed movable-suppressed channel–spatial attention mechanism integrates probabilistic movable object masking into the feature encoding process. Extensive experiments on multiple benchmarks demonstrate the effectiveness of the proposed approach: SGMR-LPR consistently outperforms both classical and learning-based baselines in highly dynamic urban scenarios, particularly on the KITTI-360 dataset, and further exhibits strong generalization capability on NCLT and OffRoad-LPR across different LiDAR configurations and scene structures. Ablation studies validate the effectiveness of the core design choices and their contribution to robust feature learning.

Despite these advances, several limitations merit discussion. First, in heavily crowded or severely occluded scenes, the semantic segmentation branch may yield uncertain predictions that degrade the quality of probabilistic movable object masks, although the soft masking mechanism partially mitigates this risk. Second, the predefined movable object category set may not cover unseen movable types absent from the training annotations. Third, the reliance on semantic annotations during training may constrain direct deployment in environments lacking suitable labels, though the generalization results on NCLT and OffRoad-LPR indicate that this dependency is alleviated under domain shifts. Addressing these limitations, including improving computational efficiency and reducing reliance on predefined categories and annotations, constitutes important directions for future work toward long-term deployment and multi-modal sensing scenarios.

## Figures and Tables

**Table 1:** Maximum $F_{1}$ scores on raw and randomly rotated KITTI datasets.

Method	Raw KITTI				Random Rotated KITTI				Diff *
	00	02	08	Mean	00	02	08	Mean	
M2DP [15]	0.708	0.717	0.073	0.499	0.276	0.282	0.201	0.253	−0.246
SC [13]	0.750	0.782	0.607	0.713	0.719	0.734	0.546	0.666	−0.047
LI [14]	0.668	0.762	0.478	0.636	0.667	0.764	0.470	0.634	−0.002
PNV [19]	0.779	0.727	0.037	0.514	0.083	0.090	0.086	0.086	−0.428
DiSCO [31]	0.964	0.892	0.903	0.920	0.960	0.891	0.892	0.914	−0.006
BEVPlace [30]	0.979	0.900	0.894	0.924	0.979	0.900	0.894	0.924	0.000
BEVPlace++ [34]	0.983	0.905	0.923	0.937	0.975	0.912	0.928	0.938	−0.001
SSC [6]	0.951	0.891	0.940	0.927	0.955	0.889	0.943	0.929	0.002
SGPR [7]	0.820	0.751	0.750	0.774	0.772	0.716	0.678	0.722	−0.052
Locus [35]	0.957	0.745	0.900	0.867	0.944	0.726	0.877	0.849	−0.018
RINet [8]	0.978	**0.947**	0.869	0.931	0.978	**0.947**	0.869	0.931	0.000
Graph-Transformer [42]	0.941	0.945	0.951	0.946	0.941	0.945	0.951	0.946	0.000
SC_LPR [36]	0.900	0.870	0.650	0.807	0.900	0.870	0.650	0.807	0.000
SG-LPR [10]	0.980	0.918	0.898	0.932	0.969	0.913	0.880	0.921	−0.011
**SGMR-LPR**	**0.986**	0.937	**0.963**	**0.962**	**0.984**	0.934	**0.960**	**0.959**	−0.003

* Diff denotes the difference between the mean performance on the Random Rotated KITTI dataset and that on the Raw KITTI dataset. A Diff value closer to zero or positive indicates better robustness to rotational changes. Bold values indicate the best result on each sequence; underlined values indicate the second-best result. The same applies to the following tables.

**Table 2:** Maximum $F_{1}$ scores on KITTI-360 dataset.

Methods	0000	0002	0004	0005	0006	0009	Mean
M2DP [15]	0.423	0.209	0.246	0.311	0.397	0.620	0.368
SC [13]	0.831	0.771	0.811	0.843	0.834	0.851	0.824
LI [14]	0.688	0.704	0.714	0.747	0.720	0.782	0.726
PNV [19]	0.352	0.349	0.325	0.285	0.295	0.330	0.323
DiSCO [31]	0.922	0.916	0.932	0.890	0.909	0.957	0.921
SSC [6]	0.921	0.974	0.975	0.974	0.978	0.970	0.965
SGPR [7]	0.818	0.788	0.795	0.798	0.833	0.843	0.813
Locus [35]	0.908	0.871	0.896	0.858	0.878	0.966	0.896
RINet [8]	0.935	0.966	0.968	0.949	0.951	0.976	0.958
Graph-Transformer [42]	0.940	0.941	0.934	0.921	0.933	0.949	0.936
SG-LPR [10]	0.942	0.953	0.949	0.951	0.968	0.965	0.955
**SGMR-LPR**	**0.972**	**0.975**	**0.980**	**0.977**	**0.982**	**0.982**	**0.978**

**Table 3:** Recall@1 on the NCLT dataset for generalization performance evaluation.

Methods	00 *	01	02	03	04	05	Mean
M2DP [15]	0.632	0.580	0.424	0.406	0.493	0.279	0.469
BoW3D [52]	0.149	0.107	0.065	0.050	0.052	0.075	0.083
CVTNet [32]	0.892	0.880	0.812	0.749	0.771	0.803	0.818
LoGG3D-Net [53]	0.699	0.196	0.110	0.087	0.109	0.256	0.243
LCDNet [54]	0.605	0.542	0.442	0.349	0.317	0.109	0.394
BEVPlace [30]	0.935	0.927	0.874	0.878	0.889	0.862	0.894
BEVPlace++ [34]	**0.953**	**0.942**	0.902	0.889	0.913	0.878	0.913
SG-LPR	0.947	0.936	0.931	0.916	0.914	0.913	0.926
**SGMR-LPR**	0.946	0.933	**0.933**	**0.930**	**0.918**	**0.943**	**0.934**

* To simplify the table, we denote the sequences “2012-01-15”, “2012-02-04”, “2012-03-17”, “2012-06-15”, “2012-09-28”, “2012-11-16”, and “2013-02-23” from the NCLT dataset as 00 to 05, respectively.

**Table 4:** Effectiveness of explicit movable object suppression measured by maximum $F_{1}$ scores on the KITTI dataset.

Method	PMOM	00	02	08	Mean
SG-LPR	✘	0.980	0.918	0.898	0.932
SGMR-LPR	✔	**0.986**	**0.937**	**0.963**	**0.962**

✘ denotes that the PMOM module is not employed; ✔ denotes that the PMOM module is employed. Bold values indicate the best result on each sequence.

**Table 5:** Ablation study on different mask types in SGMR-LPR measured by maximum $F_{1}$ scores.

Method	Mask Type	00	02	08	Mean
SGMR-LPR-MN ^1^	None	0.970	0.917	0.914	0.934
SGMR-LPR-MH ^2^	Hard	0.975	0.916	0.927	0.939
SGMR-LPR	Soft	**0.986**	**0.937**	**0.963**	**0.962**

^1^ SGMR-LPR-MN denotes the SGMR-LPR without masking. ^2^ SGMR-LPR-MH denotes the SGMR-LPR with hard masking.

**Table 6:** Ablation study on different attention types in SGMR-LPR measured by maximum $F_{1}$ scores.

Method	Attention Type	00	02	08	Mean
SGMR-LPR-cbam	CBAM	0.964	0.923	0.894	0.927
SGMR-LPR	MSCS	**0.986**	**0.937**	**0.963**	**0.962**

**Table 7:** Ablation study on attention coefficient strategies in SGMR-LPR measured by maximum $F_{1}$ scores.

Method	Coefficient	00	02	08	Mean
SGMR-LPR-learnable	Learnable	0.968	0.911	0.903	0.927
SGMR-LPR	Fixed	**0.986**	**0.937**	**0.963**	**0.962**

**Table 8:** Comparison of model parameters and computational efficiency *.

Methods	Params [M]	FLOPs [G]	Descriptor Extraction Time [ms]	Searching Time [ms]	Total Time [ms]
OT [29]	47.95	2.53	2.94	0.69	3.63
BEVPlace [30]	2.12	12.54	37.92	1.66	39.58
BEVPlace++ [34]	1.39	13.61	20.99	0.73	21.72
SG-LPR [10]	37.28	9.26	24.13	3.51	27.64
**SGMR-LPR (Ours)**	37.17	9.04	31.15	1.21	32.36

* The time values in the table represent the processing time per frame.

## Data Availability

The original contributions presented in this study are included in the article; further inquiries can be directed to the corresponding authors.

## Abbreviations

The following abbreviations are used in this manuscript:

LiDAR	Light Detection and Ranging
LPR	LiDAR-based Place Recognition
SLAM	Simultaneous Localization and Mapping
SGMR	Semantic-guided Movable-robust
BEV	Bird’s Eye View
PMOM	Probabilistic Movable Object Masking
MSCS	Movable-Suppressed Channel–Spatial
